# The relationship between retinal cone density and cortical magnification in human albinism

**DOI:** 10.1167/jov.20.6.10

**Published:** 2020-06-16

**Authors:** Erica N. Woertz, Melissa A. Wilk, Ethan J. Duwell, Jedidiah R. Mathis, Joseph Carroll, Edgar A. DeYoe

**Affiliations:** 1 Department of Radiology, Medical College of Wisconsin, Milwaukee, WI, USA; 2 Department of Ophthalmology and Visual Sciences, Medical College of Wisconsin, Milwaukee, WI, USA; 3 Department of Cell Biology, Neurobiology and Anatomy, Medical College of Wisconsin, Milwaukee, WI, USA; 4 Department of Biophysics, Medical College of Wisconsin, Milwaukee, WI, USA; 5 Present Address: Clinical Services Laboratory, HudsonAlpha Institute for Biotechnology, Huntsville, AL, USA; 6 Department of Neurology, Medical College of Wisconsin, Milwaukee, WI, USA

**Keywords:** albinism, cortical magnification, cone density, retinotopic mapping, human

## Abstract

The human fovea lies at the center of the retina and supports high-acuity vision. In normal visual system development, the highest acuity is correlated with both a high density of cone photoreceptors in the fovea and a magnified retinotopic representation of the fovea in the visual cortex. Both cone density and the cortical area dedicated to each degree of visual space—the latter describing cortical magnification (CM)—steadily decrease with increasing eccentricity from the fovea. In albinism, peak cone density at the fovea and visual acuity are decreased, but seem to be within normal limits in the periphery, thus providing a model to explore the correlation between retinal structure, cortical structure, and behavior. Here, we used adaptive optics scanning light ophthalmoscopy to assess retinal cone density and functional magnetic resonance imaging to measure CM in the primary visual cortex of normal controls and individuals with albinism. We find that retinotopic organization is more varied among individuals with albinism than previously appreciated. Additionally, CM outside the fovea is similar to that in controls, but also more variable. CM in albinism and controls exceeds that which might be predicted based on cone density alone, but is more accurately predicted by retinal ganglion cell density. This finding suggests that decreased foveal cone density in albinism may be partially counteracted by nonuniform connectivity between cones and their downstream signaling partners. Together, these results emphasize that central as well as retinal factors must be included to provide a complete picture of aberrant structure and function in albinism.

## Introduction

The human fovea occupies only 0.02% of the total retinal area, but is responsible for our highest acuity vision. The fovea is characterized by the excavation of inner retina, a lack of retinal vasculature, and a small region lacking rod photoreceptors. The fovea has the highest density of cone photoreceptors, as well as nonconvergent connections between these cones and their postsynaptic partners, known as the “midget” system (Curcio & Allen, 1990; Dacey, 1993). This leads to cortical sampling of foveal cones that is up to 160 times greater than that of peripheral cones (Duncan & Boynton, 2003), such that approximately 40% of the primary visual cortex (V1) is dedicated to the fovea (Hendrickson, 2005). Cone density decreases with increasing eccentricity, with the steepest decline occurring within 1 to 2 mm of the fovea (Curcio, Sloan, Kalina, & Hendrickson, 1990). Likewise, in V1 the amount of cortical space devoted to each degree of visual angle is magnified at the fovea and decreases steadily with increasing eccentricity (Cowey & Rolls, 1974; Daniel & Whitteridge, 1961). The retinotopic organization of V1 is believed to develop according to a two-step process, in which the gross map of visual space is first formed by experience-independent molecular guidance cues, then later refined by patterned retinal activity (Huberman, Feller, & Chapman, 2008; McLaughlin, Torborg, Feller, & O'Leary, 2003; Xu et al., 2011). Retinotopic mapping and foveal magnification in visual cortex are present even in early blind individuals, demonstrating that the gross organizational pattern is largely independent of visual experience (Bock et al., 2015; Fine & Park, 2018). The total surface area of the calcarine sulcus and banks (where V1 is located) is also believed to be strongly influenced by genetic (i.e., experience-independent) factors (Strike et al., 2019).

Behaviorally, the relationship between cortical magnification (CM) and eccentricity is thought to be directly correlated with visual acuity. Daniel & Whitteridge (1961) showed that CM in nonhuman primates roughly correlated with visual acuity thresholds in humans. This correlation was later confirmed by empirical measurements of CM in humans using electrodes implanted in the occipital lobe (Cowey & Rolls, 1974) and by functional magnetic resonance imaging (fMRI) (Popovic & Sjöstrand, 2001). Later, Duncan and Boynton (2003) used fMRI to measure CM and compared it with acuity thresholds measured in the same individuals and obtained a similar result. They concluded that CM is a fundamental neurophysiological factor limiting human visual acuity. However, recent evidence in amblyopia suggests that changes in visual acuity in some populations may not necessarily be reflected in CM, but rather that acuity may be more directly related to population receptive field size (Clavagnier, Dumoulin, & Hess, 2015). Additionally, it should be noted that some variation in fMRI-based CM measurements can reflect stimulus design and methodology (Duncan & Boynton, 2003; Engel, Glover, & Wandell, 1997; Popovic & Sjöstrand, 2001; Qiu et al., 2006; Sereno et al., 1995), which will not be mirrored in acuity thresholds.

Although methodological differences may contribute to the variation in the empirical measurement of CM, it is also possible that some variation comes from anatomical differences between individuals. In the retina, histologic studies show more than a three-fold difference in foveal cone density across individuals (Curcio et al., 1990), and the sizes of the optic tract, lateral geniculate nucleus (LGN), and V1 are known to be correlated within individuals (Andrews, Halpern, & Purves, 1997). Foveal magnification in the visual cortex has typically been thought to reflect the higher cone density and higher retinal ganglion cell (RGC) density at or near the fovea (Popovic & Sjöstrand, 2001). Consequently, it has been a working hypothesis that variations in CM across participants might be expected to reflect individual variations in cone density (Dougherty et al., 2003). Recent advances in retinal imaging with adaptive optics have made it possible to resolve the cone mosaic in the living human eye, providing the opportunity to assess cone density and CM in the same individual. Such comparisons can determine whether variation in retinal structure correlates with variation in CM.

Certain diseases affecting the visual system can lead to even greater anatomical variability between individuals than is normally observed. One example is albinism, a family of genetic diseases that disrupt melanin synthesis and trafficking, resulting in abnormal development of the visual system (Creel, O'Donnell, & Witkop, 1978; Nettleship, 1909; O'Donnell, King, Green, & Witkop, 1978). In albinism, peak cone density is lower on average than that observed in normal controls, but in some affected individuals it is within the normal range (Wilk et al., 2014). Thus, albinism is an advantageous model to probe structural correlates of variability in CM. Albinism is also characterized by aberrant decussation of optic nerve fibers at the optic chiasm. Normally, nerve fibers from the nasal retina decussate to the contralateral thalamus and fibers from the temporal retina project to the ipsilateral thalamus. In albinism, many of these temporal fibers instead project contralaterally, leading to aberrant, partially overlapped representations of opposite hemifields in visual cortex (Ather et al., 2019; Bridge et al., 2014; Hoffmann, Tolhurst, Moore, & Morland, 2003; Morland, Baseler, Hoffmann, Sharpe, & Wandell, 2001; Neveu, von dem Hagen, Morland, & Jeffery, 2008; von dem Hagen, Hoffmann, & Morland, 2008; von dem Hagen, Houston, Hoffmann, Jeffery, & Morland, 2005; von dem Hagen, Houston, Hoffmann, & Morland, 2007). Although many studies have assessed cortical reorganization in albinism, to our knowledge no studies in this population have assessed CM and its quantitative relationship to cone density. In light of these relationships between various retinal and central visual system structures, it is of particular interest to determine how CM in albinism compares with that in normal individuals and whether it correlates with observed decreases in cone density at different eccentricities. Additionally, individuals with albinism are known to experience decreased visual acuity compared with normal individuals (Summers, 1996; Wilson et al., 1988), but their visual acuity does not seem to be closely correlated with peak cone density (Wilk et al., 2014). Thus, the physiological source of acuity deficits in albinism remains unclear. Because CM may be a limiting factor for visual acuity thresholds, examining the CM in these patients could provide a bridge between visual system structure and aberrant acuity functions in albinism, as well as insight into the nature of retinocortical relationships in disease.

Here, we performed high-resolution retinal imaging using adaptive optics scanning light ophthalmoscopy and retinotopic mapping in the visual cortex using fMRI to examine both cone density and CM in the normal and albinotic visual system. We test the hypothesis that CM is decreased in participants with albinism in proportion to their unique pattern of decreased cone density versus visual field eccentricity. However, we found that CM is similar (on average) in albinism to that in controls, although it is also significantly more variable. Our results suggest that individual variations in cone density do not accurately predict CM, particularly near the fovea. Conversely, a model that accounts for convergence and divergence between cones and RGCs provides a better prediction of empirical CM estimates. Accordingly, we also test the alternate hypothesis that CM is decreased in proportion to the (estimated) pattern of ganglion cell receptive field density versus eccentricity. Our results clearly indicate that postreceptoral factors are required to account for the cortical patterns of CM within the albinism population.

## Methods

### Participants

Six participants with albinism (4 females, 2 males; aged 15–31 years) with minimal nystagmus and five participants with no prior ocular or cortical pathology (2 females, 3 males; aged 20–25 years) were recruited for this experiment. One participant with albinism was excluded from further analysis owing to significant fMRI motion artifacts (male, age 15 years); participants included in the analysis are listed in Table 1. Retinal features of these participants have been previously described (Wilk et al., 2014; Wilk, Wilk, Langlo, Cooper, & Carroll, 2017). The study was conducted in accordance with the Declaration of Helsinki and approved by the Institutional Review Board of the Medical College of Wisconsin. All participants provided written consent after explanation of the nature and possible consequences of the study.

**Table 1.** Participant demographics. Notes: *Cone density for this participant was previously reported by Wilk et al. (2014). **Cone density for this participant was previously reported by Wilk et al. (2017).

Condition	Participant	Sex	Age	Eye	Axial length(mm)	FovealCone density (cones/mm^2^)
Control	JC_0200	M	25	OD	24.72	128,560^*^
	JC_0677	F	24	OD	24.03	165,080^*^
	JC_0769	F	21	OD	24.36	127,830^*^
	JC_0905	M	20	OD	22.46	125,640^*^
	JC_0914	M	24	OD	27.98	84,730^*^
Albinism	JC_0492	F	31	OD	23.53	81,810^*^
	JC_0493	F	23	OD	22.33	89,120^*^
	JC_10093	M	19	OD	21.28	50,400^**^
	JC_10227	F	19	OD	22.82	78,890^**^
	JC_10230	F	19	OS	20.15	46,020^**^

### Fixation testing

Fixational stability was assessed in all participants with albinism using the fixation test module on the OPKO combined scanning laser ophthalmoscope (SLO) and optical coherence tomography imaging system. After the SLO scanner was focused on the participant's retina, the operator specified a group of inner retinal blood vessels to track for the duration of the run. The participant was instructed to fixate on a small white cross during the run while minimizing blinks. Participants completed three 20-second runs. Subsequently, the reference SLO images used in each run were manually registered to each other using only translation and rotation in Adobe Photoshop CS6 (Adobe Systems, Inc., San Jose, CA). The transform for each SLO image was then applied to the corresponding fixation coordinates, and the fixation points from all runs were combined to calculate the 50% and 95% bivariate contour ellipse area (BCEA). The BCEA is an elliptical area on the retina where each participant fixates, either 50% (BCEA_50_) or 95% (BCEA_95_) of the time measured. The total area of each ellipse was calculated using the following equations:

(1) $$BCEA_{50}=\;1.38\pi \sigma_{H}\sigma_{V}{\left(1-\rho^{2}\right)}^{\frac{1}{2}}$$

(2) $$BCEA_{95}=\;6.00\pi \sigma_{H}\sigma_{V}{\left(1-\rho^{2}\right)}^{\frac{1}{2}}$$

where *σ*_H_ and *σ*_V_ are the standard deviations (SDs) of the coordinates in the horizontal and vertical directions, respectively, and *ρ* is the product-moment correlation of the horizontal and vertical coordinates (Crossland, Sims, Galbraith, & Rubin, 2004; Steinman, 1965).

### Retinal imaging

The axial length of the eye (used for lateral scaling of retinal images) was obtained for each participant using an IOL Master (Carl Zeiss Meditec, Dublin, CA). One eye for each participant was dilated and accommodation was suspended using one drop each of phenylephrine hydrochloride (2.5%) and tropicamide (1%). A previously described adaptive optics scanning light ophthalmoscope (Dubra & Sulai, 2011) was used to obtain images of the photoreceptor mosaic at the fovea and a strip in the temporal retina. To produce an image with minimal distortion, raw videos were first “desinusoided” to correct for the sinusoidal motion of the resonant scanner by estimating the distortion from images of a Ronchi ruling and then resampling the images over a grid of equally spaced pixels (Cooper et al., 2011). The videos were then manually inspected for reference frames that contained minimal distortion, which were then used for image registration using custom software (Dubra & Harvey, 2010). Registered images were manually aligned using Adobe Photoshop (Adobe Systems, Inc.). Foveal cones were identified using a previously described, semiautomated algorithm (Garrioch et al., 2012). The location of peak cone density was identified and cone density was measured at locations across the temporal retina using a 37-μm sampling window as previously described (Wilk et al., 2014). For images within approximately 2° of the fovea, cones were counted using the same semiautomated algorithm (Garrioch et al., 2012). For peripheral images, cones were identified manually by a single observer (MAW) using ImageJ (Schneider, Rasband, & Eliceiri, 2012) or custom Java software (Oracle Corporation, Redwood Shores, CA) (Cooper, Wilk, Tarima, & Carroll, 2016).

### Functional MRI visual stimuli

All fMRI stimuli were presented on a back-projection screen mounted on the MR head coil using a BrainLogics BLMRDP-A05 MR digital projector (Puckett & DeYoe, 2015). Stimuli were generated using a ViSaGe MKII visual stimulus generator (Cambridge Research Systems, Rochester, United Kingdom) in conjunction with MATLAB.

Stimuli included conventional expanding ring and rotating wedge retinotopic mapping stimuli (DeYoe et al., 1996). Rings and wedges were composed of black and white counterphase flickering circular checkerboards (8 Hz) with check size and ring width scaled with eccentricity. Stimuli were presented on a uniform gray background and subtended a maximum of 20° eccentricity. All participants were instructed to continually fixate at the center of the screen. To enhance fixational stability, thin, black radial lines extending from fixation to the edge of the display were present continuously in all tasks.

To minimize unnecessary duplication, previous retinotopic mapping data for normal control participants were obtained using slightly different stimulus parameters than for the participants with albinism. However, owing to the temporal phase mapping methods used in this study, these differences did not significantly affect our results. For participants with albinism, the wedge stimuli subtended 45° polar angle, and for control participants the wedges subtended 90°. All participants viewed full-field ring and wedge stimuli binocularly. Additionally, for participants with albinism, the expanding ring stimuli were presented to the right and left hemifields in separate runs and were tested separately for each eye. Hemifield ring stimuli were identical to the full-field versions, except that one hemifield was masked to match the grey background. For control participants, the ring stimulus expanded from the center to the periphery in 40 seconds and was repeated five times per run with a 2-second interval between cycles of expanding rings. For participants with albinism, both the full-field and hemifield ring stimuli expanded from 0.8° eccentricity to the periphery in 60 seconds and were repeated five times per run with a 7-second interval between cycles of expanding rings. For participants with albinism, the center of the display consisted of a circular black and white disc (similar to a radioactivity symbol) with a radius of 0.8° that appeared and flickered at random intervals not synchronized to the rings and wedges presentation. To control attention, participants were instructed to press and hold a button whenever the central disc appeared and flickered.

### Functional MRI stimulus paradigm

Control participants completed all imaging during a single session. Participants with albinism completed imaging during two sessions: the right eye hemifield expanding ring tasks in the first session and all remaining tasks in the second session. All monocular hemifield runs were repeated five times (with one exception: for JC_10230, monocular hemifield runs were repeated three times with right eye viewing and four times with left eye viewing) and binocular full-field runs were repeated three times. For monocular stimuli, repetitions of the right and left hemifield stimuli were interleaved; for full-field stimuli, repetitions of the expanding ring and rotating wedge were interleaved. After each fMRI run, the participant was asked to rate their alertness on a scale from 1 to 5 (1 being asleep and 5 being fully awake). Participants’ alertness can affect the quality of data, so this measure was included as a potential exclusion criterion. No data were excluded from this study based on the alertness ratings.

### Functional MRI acquisition

Scans were completed using a 3.0 Tesla General Electric Signa Excite 750 MRI system at the Medical College of Wisconsin. A custom 32-channel RF/Gradient head coil and a T2*-weighted gradient-echo EPI pulse sequence (TE = 25 ms, TR = 2 s, FA = 77°) were used. The 96 × 96 acquisition matrix (Fourier interpolated to 128 × 128) had frequency encoding in the right–left axial plane, phase encoding in anterior–posterior direction, and slice selection in the axial direction. The field of view was 240 mm and included 29 axial slices in the occipital lobe and adjacent portions of the temporal and parietal lobes with a slice thickness of 2.5 mm, yielding a raw voxel size of 2.5 mm^3^. The data were Fourier interpolated to 1.875 × 1.875 × 2.5 mm. For anatomical scans, a T1-weighted spoiled gradient recalled (SPGR) echo-planar at steady state pulse sequence was used (TE = 3.2 ms, TR = 8.2 ms, FA = 12°) with a 256 × 224 acquisition matrix (Fourier interpolated to 256 × 256). The field of view was 24 cm, and 180 slices with a slice thickness of 1.0 mm, yielding raw voxel sizes of 0.938 × 1.07 × 1.0 mm^3^. The SPGR scans were Fourier interpolated to 0.938 × 0.938 × 1.0 mm^3^ and subsequently resampled to 1.0 mm^3^. A sync pulse from the scanner at the beginning of each run triggered the onset of visual stimuli.

### Analysis software

All fMRI data were analyzed using the AFNI/SUMA package (Cox, 1996). Surface models were produced from the high-resolution SPGR images using Freesurfer (version 5.1.0 or 5.3.0, http://surfer.nmr.mgh.harvard.edu/) with the recon-all function.

### Functional MRI preprocessing

For all participants except two (JC_10093 and JC_10227, discussed elsewhere in this article), fMRI preprocessing was performed in the following order: reconstruction, volume registration, averaging of the time courses, removal of the initial magnetization transients, and alignment. Volumes from all individual runs in an interleaved block were registered to the middle volume of the first run in the block using AFNI 3dVolreg. Individual runs for each functional task were averaged using AFNI 3dMean to produce average time courses. The BEFORE and AFTER periods were removed using AFNI 3dcalc, and the averaged time series data were then aligned to the reference anatomical scan.

For participants with albinism, we attempted to minimize bias in the alignment of functional scans from either of the two sessions to the reference anatomy by using a modified version of the align_across_days.csh script available on the AFNI and NIfTI server (https://sscc.nimh.nih.gov/sscc/dglen/alignmentacross2sessions). In this script, the reference SPGR anatomical images from both sessions were skull-stripped using AFNI 3dSkullStrip, aligned using AFNI align_epi_anat.py, and averaged using AFNI 3dMean to create an average reference anatomy for the two sessions. Average functional runs were then aligned to these average reference anatomies using align_epi_anat.py.

For control participants, all data were acquired in a single session, so the creation of an average reference anatomy was unnecessary. All preprocessing was otherwise the same, and functional runs for control participants were aligned to reference SPGR anatomical scans using the same align_epi_anat.py command.

Participants JC_10093 and JC_10227 had greater head movement between runs, so a modified preprocessing pipeline was used that provided better volume registration. The modified preprocessing was performed in the following order: reconstruction, volume registration, alignment, averaging of the time courses, and removal of the initial magnetization transients. For volume registration, a mean reference volume was calculated for each task by aligning and averaging the middle volume of each individual run. All volumes in each run were then registered to this average volume using AFNI 3dVolreg. Each run was then aligned to the average reference anatomy, averaged, and had BEFORE and AFTER periods removed using the AFNI functions as described.

### Phase-encoded retinotopic maps

Phase-encoded retinotopic activation maps were generated to plot the spatial distribution of fMRI responses corresponding to the visual field eccentricities or polar angles that were stimulated in each of the functional tasks. Significant responses were identified by cross-correlating the empirical time course data for each voxel with a reference waveform using AFNI 3ddelay (Bandettini, Jesmanowicz, Wong, & Hyde, 1993; Datta & DeYoe, 2009; Saad, DeYoe, & Ropella, 2003). This analysis produces the correlation coefficient and temporal delay at the phase offset of maximum correlation for each voxel. The reference waveform used for this phase mapping procedure was a binary square wave describing the stimulus cycles convolved temporally with the “Cox Wide” estimation of the hemodynamic response function. Time courses were spatially smoothed with a 3.75 mm spherical kernel using AFNI 3dLocalstat prior to this phase mapping procedure and all functional runs were thresholded to a minimum correlation coefficient of 0.30. Phase-mapped eccentricity and polar angle values at each voxel were then projected onto functional field maps (FFMaps) using Prism View (Version 4.1.0; Prism Clinical Imaging, Elm Grove, WI) as previously described (Reitsma et al., 2013), and the eccentricity and polar angle FFMaps were manually calibrated (expanded or rotated, respectively) so as to accurately represent the corresponding visual stimulus parameter. The correction factor used to make this adjustment, expressed as a constant temporal “offset” (in seconds), was then applied to the temporal delay values for all voxels in subsequent analyses.

### V1 surface area

**Figure 1.**
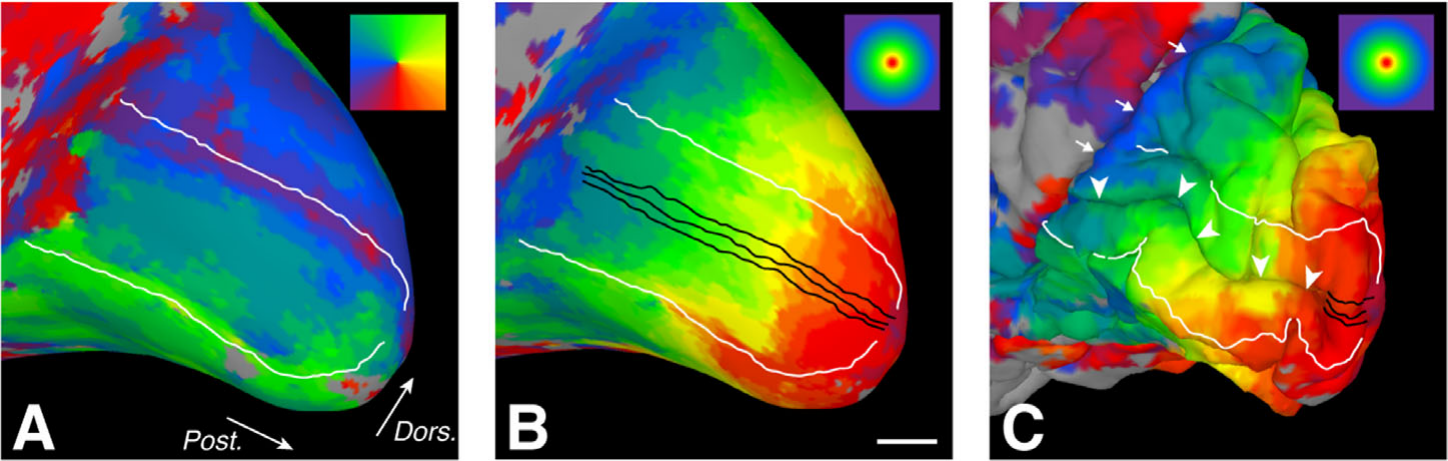
Medial occipital lobe retinotopic maps and sampling ROIs displayed on the cortical surface model for a representative control participant. Color coding for polar angle and eccentricity are shown in the upper-right corner of panels A and B/C, respectively. (A) V1/V2 boundaries (white) associated with representations of the superior and inferior vertical meridia (green and purple-red, respectively). White arrows indicate posterior (Post.) and dorsal (Dors.) orientation and apply to all panels. (B) ROIs (black) used to compute cortical mapping functions oriented parallel to the representation of the horizontal meridian within the calcarine sulcus (not shown). Phase-encoded eccentricity values (surface coloring) were assigned to each node along the linear ROIs. Scale bar = 10 mm, but is approximate owing to known distortions in cortical distance on the inflated surface. (C) Linear distances along the ROIs (buried within the calcarine sulcus) were calculated from the pial surface of the original folded three-dimensional surface model (not inflated). White arrowheads indicate the calcarine sulcus. White arrows indicate the parieto-occipital sulcus.

Using polar angle fMRI maps on the inflated cortical surface, V1 boundaries were defined by the superior (ventrally) and inferior (dorsally) vertical meridian representations along the banks of the calcarine sulcus (Figure 1A). Both 4° and 16° isoeccentricity boundaries from the fMRI eccentricity map of V1 were marked manually on inflated cortical surfaces. These boundaries were then connected to the ventral and dorsal boundaries of V1 so as to define regions of interest containing all surface nodes within each eccentricity boundary. Surface area of V1 within these ROIs was calculated using the AFNI SurfMeasures function. Surface area was calculated at both the pial surface and the gray/white matter boundary, then averaged to approximate layer four of the striate cortex. All measurements were then corrected for global brain size using the sum total surface area of the two hemispheres as calculated in Freesurfer (Aguirre et al., 2016). However, one control participant (JC_0769) and two participants with albinism (JC_0492 and JC_0493) had signal dropout in the superior region of the anatomical scans that prevented accurate segmentation of the global brain surface area. For JC_0492 and JC_0493, anatomical scans acquired on different days were used to measure global surface area. For JC_0769, the average surface area of the remaining control participants was used as a surrogate for this participant's global brain size. For statistical comparison between groups, the left and right hemispheres for each participant were added together.

### CM function modeling

Three linear ROIs were drawn on inflated cortical surfaces along and parallel to the horizontal meridian representation within V1 for each participant (Figure 1B). Using the volumetric functional data (Figure 1C), phase-encoded eccentricity and/or polar angle within the visual field was assigned to each surface node using AFNI vol2surf and custom Matlab software. The eccentricity for each node was then matched to its linear distance along each ROI using pial (i.e., noninflated) surface measurements in AFNI (Figure 1C). The total distance along each ROI was corrected by a factor of 0.93 to account for the small random variations in position of nodes in the cortical surface mesh relative to the ROIs drawn on a smooth inflated surface (correction factor determined by a previous analysis comparing mesh-based ROIs with directly measured distances). Using custom Matlab software, empirical cortical mapping functions were created by plotting the visual field eccentricity represented by each voxel against its corrected distance along the ROI (i.e., voxel location in the cortex). Although our mapping data extended to 20°, all points beyond 16° were excluded from further analysis because large population receptive field sizes in the periphery can introduce measurement artifacts in the shape of cortical mapping curves (Baseler et al., 2002). Additionally, in JC_10230 the ROIs from one hemisphere in one task were cropped at the beginning of the ROIs to eliminate a few highly aberrant measurements that prevented accurate curve fitting.

The cortical mapping data were then fit with a previously described exponential curve (Engel et al., 1997), in which eccentricity in visual space (in degrees), *E*, was modeled as a function of cortical distance (in millimeters), *d*:

(3) $$\begin{array}{c} E\left(d\right)=e^{c*\left(d+d_{0}\right)}\;\;\;\;\; \end{array}$$

In this model, the parameter *c* is a “map scaling factor” that determines the overall shape of the function. First, this curve was fit to each individual ROI using the fminsearch function in Matlab. Next, to facilitate aligning the data from the three separate ROIs to each other, the distance along each ROI (*d*) was expressed as an offset from the location representing 8° eccentricity (i.e., when *d* = 0 mm, *E(d)* = 8°). Finally, all of the data points from all three ROIs were plotted together and a single curve was then fit to them. This provided an “average” cortical mapping function based on all three ROIs. For all participants, each hemisphere in each task was modeled separately.

The fitted cortical mapping function was then used to derive a linear CM function (CMF). First, we note that linear CM refers to the number of millimeters of cortex devoted to each degree of visual space (∆*mm*/∆*degrees*), which varies with eccentricity. The empirical cortical mapping function, on the other hand, relates visual field eccentricity (degrees) to cortical distance (mm), where the slope is effectively ∆*degrees*/∆*mm*. So, if we invert this function by swapping axes, we get a function in which cortical distance (mm) is plotted against visual field eccentricity (degrees). The slope of this function (∆*mm*/∆*degrees*)—that is, the derivative—at each point then gives us the values we seek: CM. In sum, the CMF was computed as the derivative of the inverted cortical mapping function. The CMF was computed piecewise using custom Matlab software, but can be represented analytically (Qiu et al., 2006) as:

(4) $$\frac{dd}{dE}=\frac{1}{c*E}\;$$

Analogous to the role of *c* in Equation 3, 1/*c* is effectively a “CMF scaling factor” that determines the shape of the CMF.

Our goal then, is to determine if the shape of the CMF derived from empirical cortical retinotopic data are consistent with a theoretical CMF determined solely by cone density with no differential convergence or divergence in the connections of those cones at different eccentricities. Accordingly, the linear cone photoreceptor density function, *P*(*E*), for each participant was used to calculate a *predicted* cortical mapping function based on the assumption that each cone (*p*) is represented by an equal distance (∆*d*) in V1. Our use here of *P*(*E*) as linear cone density in our calculation should not be confused with areal cone density (e.g., as in Table 1), which is sometimes used in calculations in other publications. Here, *P*(*E*) is the square root of areal cone density. Thus, the mapping of retinal eccentricity (*E*) to cortical linear distance (*d*), given the aforementioned assumption, was computed as:

(5) $$\begin{array}{c} d\;=\;\int_{0}^{E}P\left(E\right)*\Delta d_{p}\;\;\;\; \end{array}$$

In other words, the integral of *P*(*E*) from the fovea (0 degrees) to a given eccentricity (*E*) yields the number of cones spanning that eccentricity range. If this number is then multiplied by the incremental distance in the cortex allotted to each cone (∆*d_p_*), the result is the distance in the cortex allotted to the eccentricity range 0 to *E* (degrees of visual angle). This can be represented graphically by plotting cortical distance (mm) as a function of visual field eccentricity (degrees) with a slope expressed in millimeters per degree. Inverting this function (i.e., swapping the axes) yields a predicted cortical mapping function with a slope expressed in ∆*degrees*/∆*mm*. A predicted CMF was then computed as the derivative of the predicted cortical mapping function in the same manner as described elsewhere in this article for the empirical cortical mapping function. The ∆*d_p_* factor (i.e., the incremental linear cortical distance allotted to each cone) was implicitly computed so as to evenly distribute the cumulative cone count from 0° to 16° over the cortical ROI distance subtending the same eccentricity range.

Finally, we made another prediction of the cortical mapping function that was based on RGC density rather than cone density. This was computed in a similar manner as above, but began with the RGC receptive field density (*G*) versus eccentricity (*E*). In this case, the prediction is based on the assumption that each RGC receptive field (*g*) was represented by an equal distance in V1. The *G* was calculated for each participant using their measured cone densities and the cone: RGC ratios reported by Drasdo, Millican, Katholi, and Curcio (2007). Like cone densities, the cone: RGC ratios reported by Drasdo et al. (2007) vary with retinal eccentricity. Although these ratios also likely vary to some degree across participants, we have only one estimate from the study by Drasdo et al. (2007), so have used their data for all our participants. The prediction then proceeds as described elsewhere in this article:

(6) $$\begin{array}{c} d\;=\;\int_{0}^{E}G\left(E\right)*\Delta d_{g}\;\;\;\; \end{array}$$

Again, inverting this function provided a predicted cortical mapping function, from which a predicted CMF was computed. Note that in this case it is not appropriate to use the density of ganglion cell bodies owing to the spatial displacement of ganglion cell bodies relative to their receptive field position in retinal visual space. This physical displacement is reflected in the length of Henle fibers and is maximal close to the fovea (Drasdo et al., 2007).

### Statistical analyses

All statistical comparisons were performed using Prism 8 (GraphPad Software, Inc.). The Shapiro-Wilk test was used to assess normality, and data were classified as normal when *p* > 0.05. Parametric tests (two-tailed unpaired and paired *t*-tests) were used to compare normal data, and nonparametric tests (Mann-Whitney *U* test and Wilcoxson matched-pairs test) were used to compare non-normal data or those with significantly different variance between groups. Differences between groups were considered to be significant when *p* < 0.05.

## Results

### Fixational stability

The 50% and 95% BCEA for each eye in each participant with albinism are shown in Table 2. Participants had 50% BCEAs ranging from 0.04 to 5.97 deg^2^ with an average of 1.40 deg^2^. The 95% BCEAs ranged from 0.18 to 25.97 deg^2^ with an average of 6.07 deg^2^. Previously, “steady fixation” has been defined as having 50% of fixation points fall within a 2°-diameter circle (i.e., 3.14 deg^2^ area) centered at the locus of fixation (Fujii et al., 2003). The BCEA reported here is elliptical (rather than circular) because eye movements tend to be predominantly horizontal. As indicated by the 50% BCEAs shown in Table 2, all participants except for one (JC_10093) had 50% BCEAs that were less than 3.14 deg^2^.

**Table 2.** Fixational stability in participants with albinism. Notes: All values are expressed in degrees^2^.

	Right eye		Left eye	
Participant	50% BCEA	95% BCEA	50% BCEA	95% BCEA
JC_0492	0.04	0.19	0.06	0.28
JC_0493	0.84	3.66	0.14	0.597
JC_10093	3.68	16.01	5.97	25.95
JC_10227	0.24	1.05	0.45	1.95
JC_10230	1.92	8.35	0.62	2.68

### Cone density

**Figure 2.**
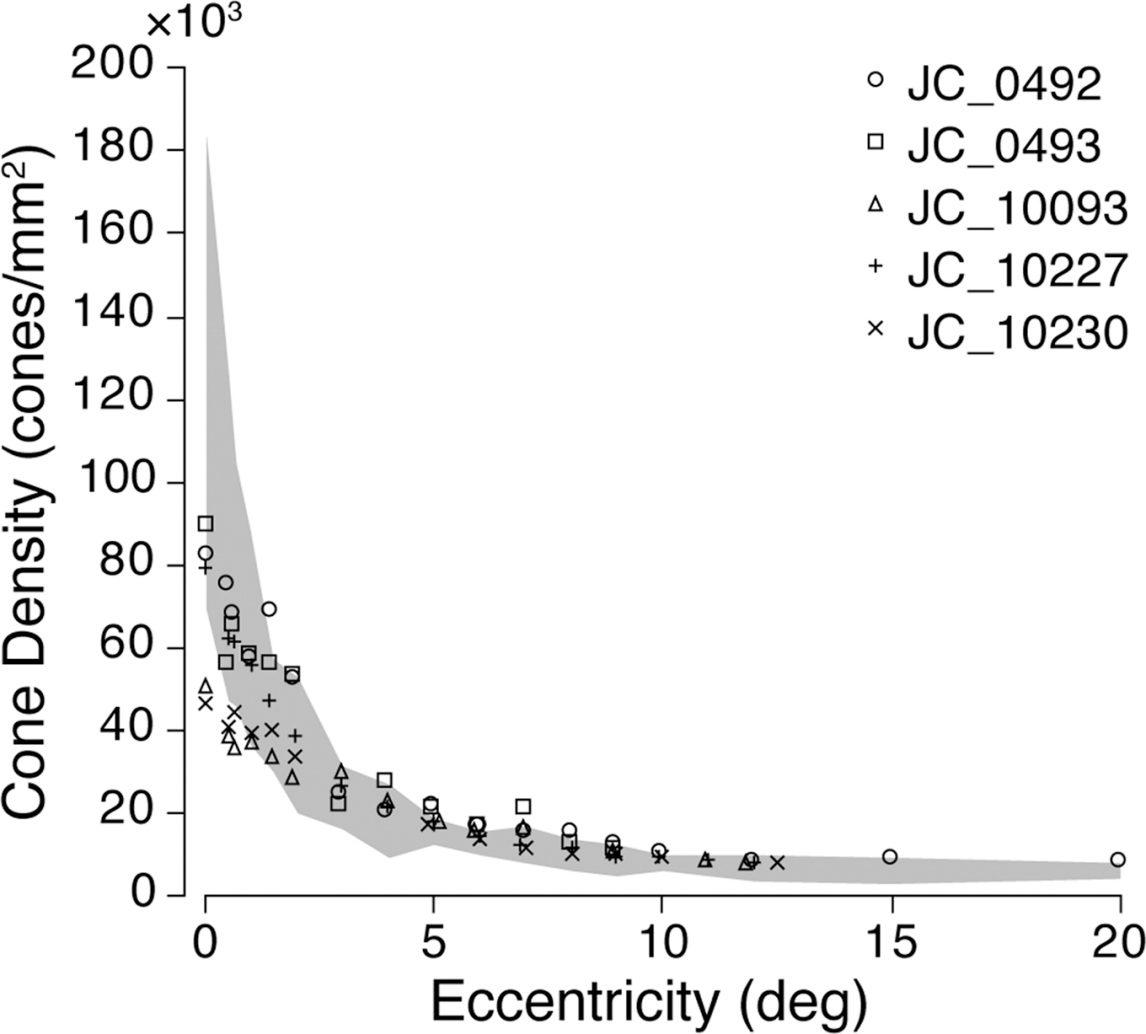
Retinal cone density as a function of eccentricity. Each participant with albinism is represented by a different data point symbol. Gray shaded area represents the average for all control participants ± 2 SD.

Control participants’ foveal peak cone densities ranged from 84,730 to 165,080 cones/mm^2^, mean ± SD: 126,370 ± 28,460 cones/mm^2^, whereas patients with albinism had foveal cone densities ranging from 46,020 to 89,120 cones/mm^2^, average ± SD = 69,250 ± 19,620 cones/mm^2^. Cone density as a function of eccentricity for each participant with albinism (symbols) and for controls (gray shading) can be seen in Figure 2. All participants showed a decrease in cone density with eccentricity. Participants with albinism generally had lower densities within 3° of the fovea; however, cone density became more similar across all participants in the periphery (Figure 2). Three participants with albinism (JC_0492, JC_0493, and JC_10227) had peak cone densities that fell within two SDs of normal, whereas the other two participants with albinism (JC_10093 and JC_10230) had peak cone densities below the normal distribution (see data points at 0° eccentricity).

### Surface area of V1

**Figure 3.**
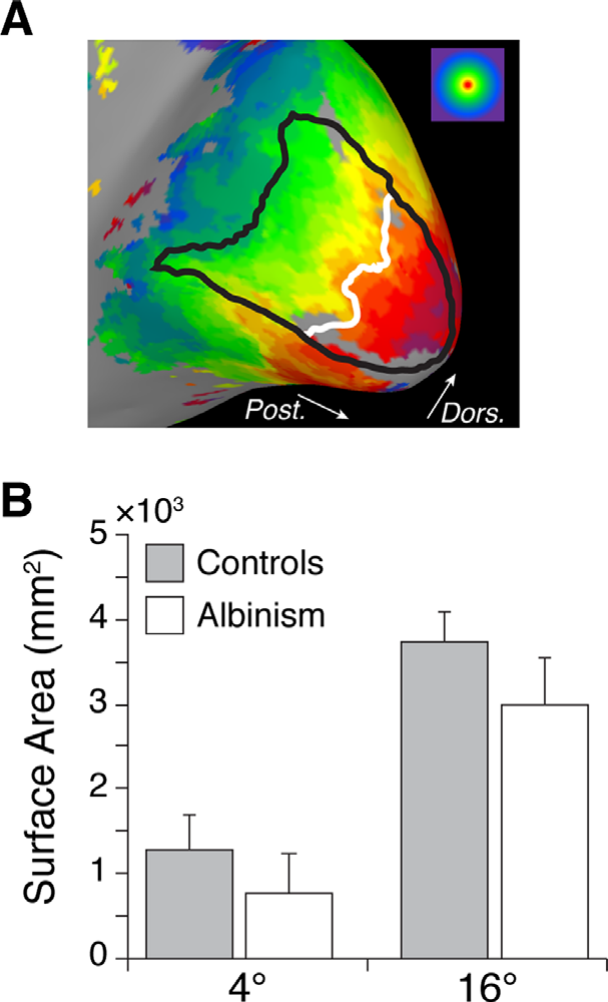
Total V1 surface area (SA) in both hemispheres within 4° and 16° eccentricity. (A) Manually drawn V1 boundary within 16° eccentricity (black) and 4° isoeccentricity contour (white) shown on the inflated medial occipital lobe surface of a representative control participant. Color coding for eccentricity is shown in the upper-right corner. White arrows indicate posterior (Post.) and dorsal (Dors.) orientation. (B) Mean V1 SA within 4° and 16° eccentricity in controls and participants with albinism. Error bars represent 1 SD. Values within 16° for participants with albinism are from only four participants owing to incompleteness of cortical maps in one participant (JC_10230). V1 SA is not significantly different between participants with albinism and controls either within 4° eccentricity, unpaired *t*-test: *t* = 2.14, df = 8, p = 0.065, or within 16° eccentricity, Mann-Whitney *U* test: *U* = 2, p = 0.064.

The cortical surface area of V1 representing the central 4° of the visual field was measured in all participants and the surface area of V1 within the central 16° of the visual field was measured in all control participants and four participants with albinism using manually drawn ROIs based on isopolar and isoeccentricity maps (Figure 3A). V1 was not measured within 16° in JC_10230 because the thresholded eccentricity data (i.e., voxels with a correlation coefficient of 0.30 or greater) did not extend all the way to 16°. Although the surface area seemed to be decreased in albinism relative to controls (Figure 3B), this difference approached but did not achieve statistical significance for either the central 4°, unpaired *t*-test: *t* = 2.14, df = 8, *p* = 0.065, or the central 16°, Mann-Whitney *U* test: *U* = 2, *p* = 0.064.

### Retinotopy in albinism

**Figure 4.**
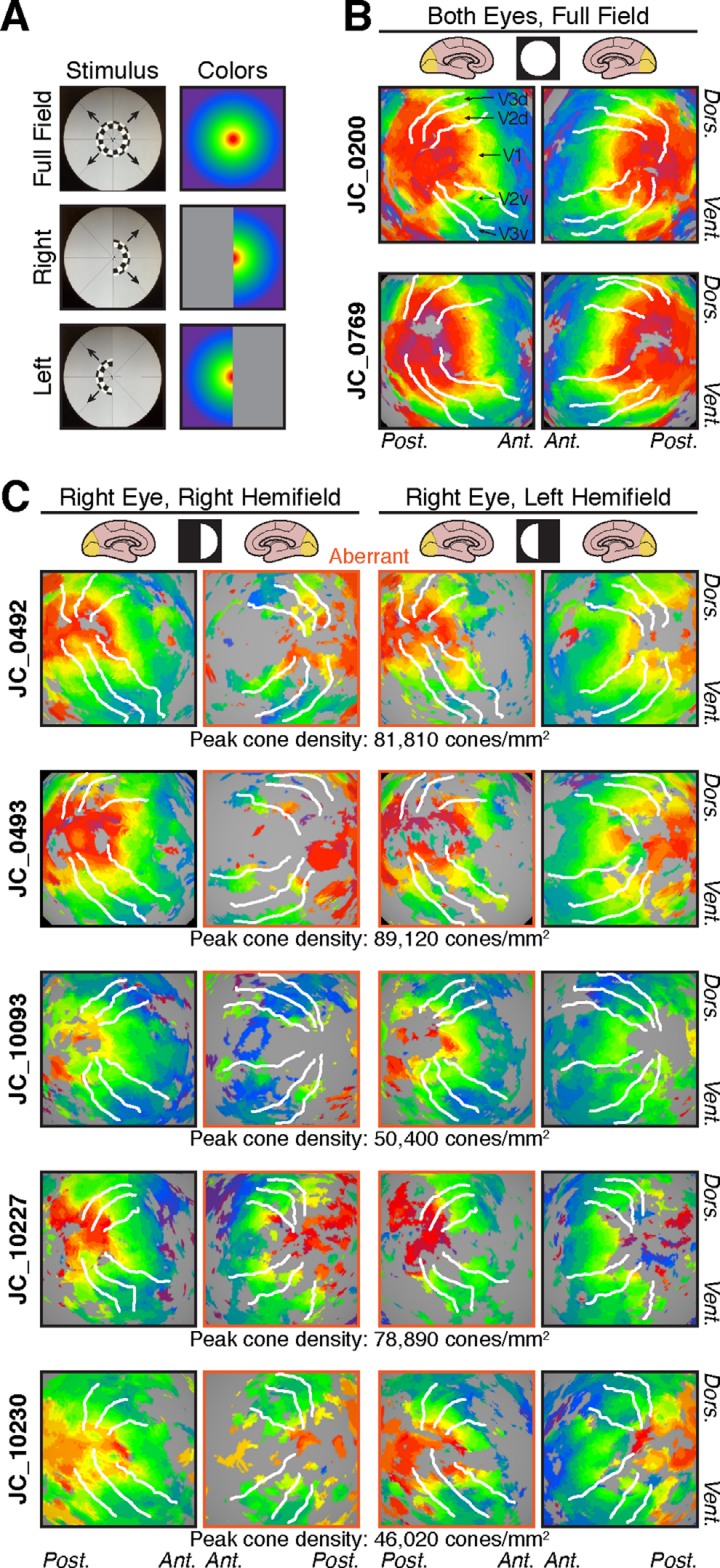
Retinotopic maps of visual field eccentricity. (A) Visual stimuli and color coding used for eccentricity mapping in controls (full field) and participants with albinism (hemifield stimuli). (B) Eccentricity maps for two representative control participants. All control participants were tested using binocular viewing of the full-field ring stimulus. (C) Eccentricity maps for participants with albinism, obtained using hemifield ring stimuli, viewed monocularly. Data for the right eye viewing condition are shown here (see Supplementary Figure S1 for left eye data). Retinotopy patterns outlined in orange (middle columns) are aberrant ipsilateral hemifield representations. Peak cone densities for each participant with albinism are indicated below each row. All retinotopic maps are displayed on spherically inflated cortical surface models. Visual stimuli are indicated by white circle/semicircle symbols at head of respective columns. White lines in B and C mark dorsal and ventral boundaries of V1/2/3 based on polar angle data (cf. Figure 1); visual areas are labeled on the left hemisphere of JC_0200 in panel (B), and labels apply for all images. Dors. = dorsal; Vent. = ventral; Ant. = anterior; Post. = posterior.

For control participants, isoeccentricity retinotopic maps were obtained using binocular, full-field stimuli (Figure 4A), which showed normal retinotopic organization in V1 (Figure 4B). For participants with albinism, retinotopic maps (Figure 4C) were acquired using monocular hemifield stimuli (Figure 4A) as described previously (Hoffmann et al., 2003). In all participants, retinotopic activation was typically most complete and contiguous when both the eye and the visual field being stimulated were contralateral to the hemisphere of interest, as would normally be expected (Figure 4C, columns 1 and 4; see also data for the left eye in Supplementary Figure S1). However, each hemisphere could also be activated by the ipsilateral visual field, which resulted in overlaid representations of both hemifields within the same hemisphere, a highly aberrant result (Figure 4C, columns 1 and 3 for the left hemisphere, columns 2 and 4 for right hemisphere). These aberrant hemifield representations were asymmetric, with the most extensive activation in the hemisphere contralateral to the stimulated eye. Thus, when the right eye was stimulated (as in Figure 4C) the aberrant activation of the left hemisphere was more prominent than that in the right hemisphere (Figure 4C, column 3 greater than column 2), and when the left eye was stimulated the aberrant activation of the right hemisphere was more prominent than that in the left hemisphere (Supplementary Figure S1, column 2 greater than column 3). This asymmetry is also evident when comparing the normal activation to the aberrant activation in the same hemisphere: for example, in participant JC_0492, when the right eye was stimulated the normal and aberrant representations in the left hemisphere (i.e., contralateral hemisphere; Figure 4C, columns 1 and 3) seemed to be more similar than the two representations in the right hemisphere (i.e., ipsilateral hemisphere; Figure 4C, columns 2 and 4). This was a general trend across participants with albinism. There was a similar trend for the left eye stimulus, with the greatest similarity between normal and aberrant representations in the right hemisphere (see Supplementary Figure S1, where columns 2 and 4 were more similar to each other than columns 1 and 3).

In addition to these trends, however, there was also significant variation across our albinism cohort (compare down each column of Figure 4C), which was particularly evident with respect to the overall completeness and continuity of the eccentricity maps. This difference can be seen clearly in participants JC_0492 and JC_10093, who showed some of the most and least contiguous maps: JC_0492 had a nearly complete retinotopic representation with few holes (especially in the left hemisphere), whereas JC_10093 showed overall more sparse activation. Additionally, there was significant variation in the relative proportions of central versus peripheral representation.

Participants in the albinism cohort also varied considerably in the predominance of central versus peripheral field representations (note the dominant colors in each participant in Figure 4C). This finding is illustrated by directly comparing participants JC_0493 and JC_10093: JC_0493 had much more central (red/orange) activation than JC_10093, but JC_10093 had greater peripheral (blue) activation than JC_0493. Additionally, participants varied in the relative spatial positions of the isoeccentricity bands: participants who had weak foveal activation also had peripheral activation that seemed to be closer to the occipital pole, where the foveal confluence is typically found (compare the location of the yellow band across participants in Figure 4C). In general, the extent of activation by the central visual field seemed to correlate with participants’ peak cone densities (shown below each row of images in Figure 4C), but JC_10230 was a notable exception. This participant had the lowest peak cone density, but had a more extensive central field representation (red) than JC_10093, who had the next lowest peak cone density. When surface area within 4° was compared with peak cone density, these measures were not significantly correlated in controls, *r*^2^ = 0.17, *p* = 0.484, or in albinism, *r*^2^ = 0.47, *p* = 0.203.

### CM in albinism

**Figure 5.**
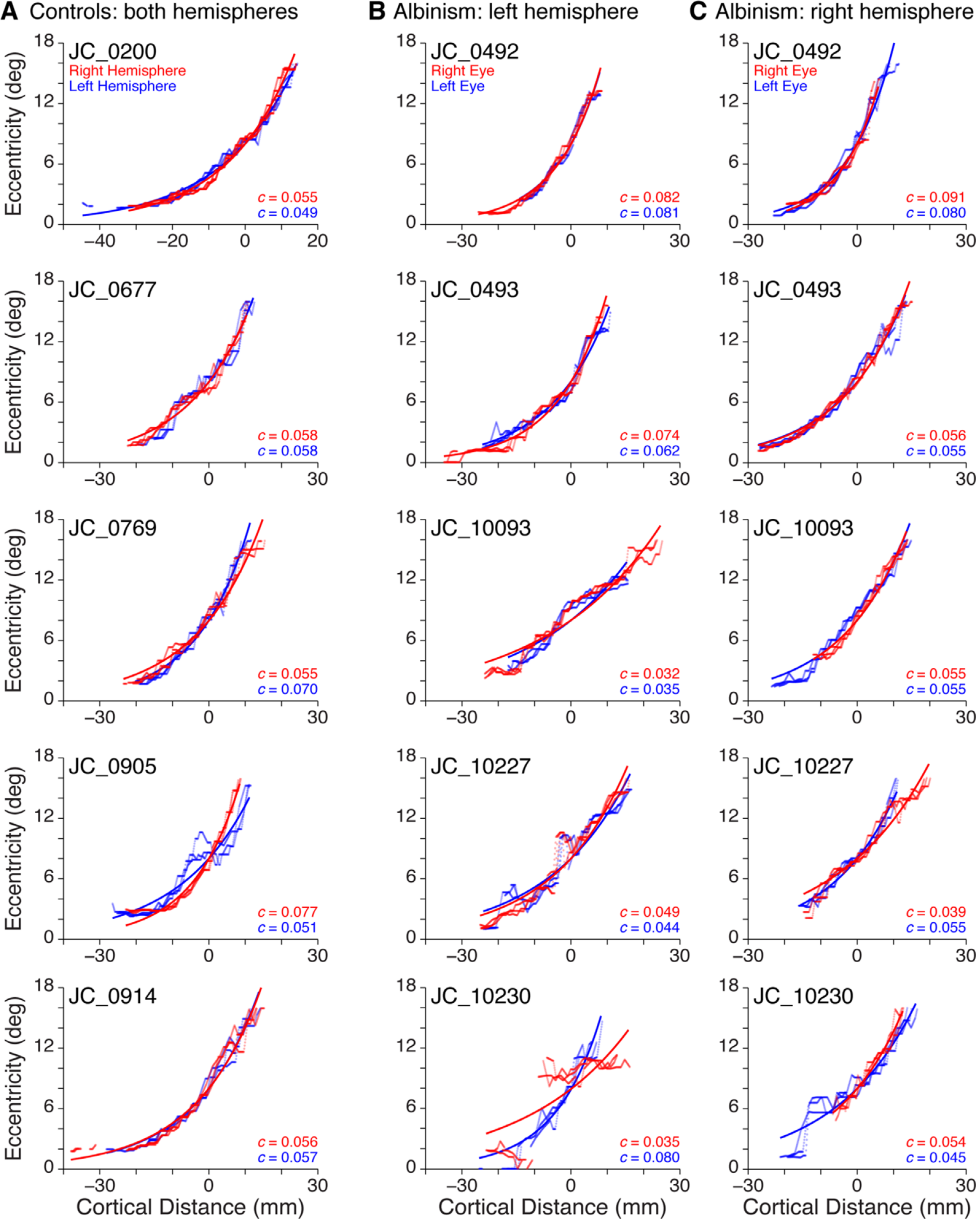
Empirical cortical mapping data and fitted functions for all participants. (A) Controls: right hemisphere (red) and left hemisphere (blue). (B) Albinism: left hemisphere, right hemifield stimulus. (C) Albinism: right hemisphere, left hemifield stimulus. Viewing conditions in B, C: right eye (red), left eye (blue). The map scaling factor for each fitted curve (Equation 3) is included in the lower-right corner of each graph.

**Figure 6.**
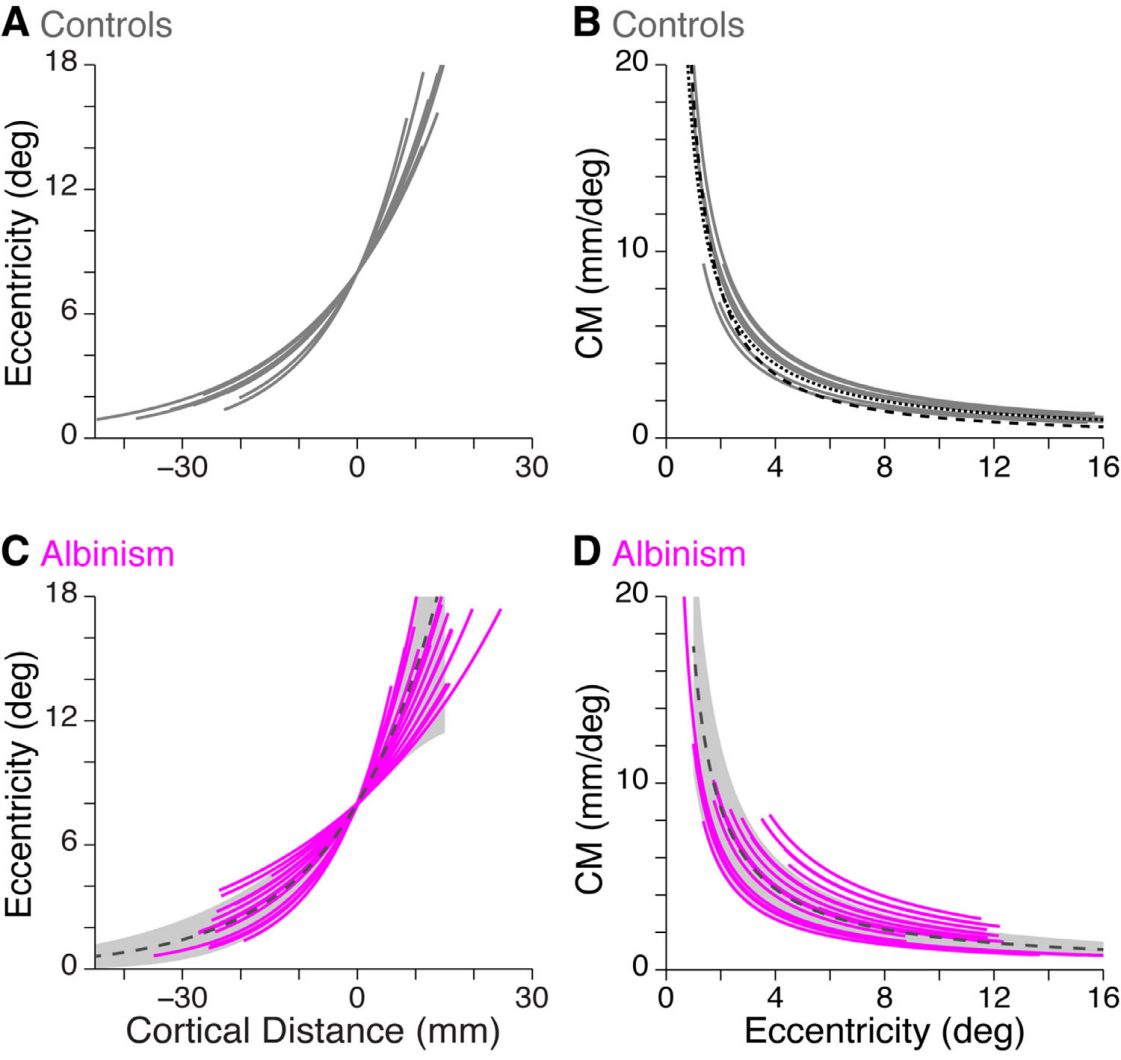
Cortical mapping and magnification functions in controls and participants with albinism. (A) Fitted cortical mapping functions and (B) corresponding CMFs (see Methods) for both hemispheres of all control participants (gray curves). In (B) the CMFs reported by Sereno et al. (1995) (dashed black curve) and by Engel, Glover, & Wandell (1997) (dotted black curve) are shown for comparison. (C) Fitted cortical mapping functions and (D) corresponding CMFs for participants with albinism (magenta curves). Dashed dark gray lines and gray shaded regions show mean ± 1 SD for controls. In C and D, data are combined for left hemisphere, right hemifield stimulus and right hemisphere, left hemifield stimulus and for both right and left eye viewing conditions.

Cortical mapping functions for all participants are shown in Figure 5. The empirical data for the three ROIs within each hemisphere are shown by the red and blue points, which appear as irregular curves (controls: red/blue = right/left hemisphere; albinism: red/blue = right/left eye). Fitted curves (Equation 3, Methods) are shown as the smooth colored lines. The fitted curves for all controls are shown in Figure 6A and for all participants with albinism in Figure 6C.

For control participants, the average ± SD map scaling factor (*c*, from Equation 3) among all hemispheres (*n* = 10) was 0.059 ± 0.009. When *c* was compared across control participant hemispheres, there was no significant difference between the right and left hemispheres, Wilcoxson matched-pairs test, *p* > 0.99.

For participants with albinism, each visual hemifield in each eye was stimulated separately, resulting in eight possible cortical mapping functions for each participant (2 hemispheres × 2 visual hemifields × 2 eyes). However, owing to the limited extent of activation by the ipsilateral hemifield in either hemisphere, Figure 5B and C and Figure 6C and D show only the curves representing activation by the contralateral visual hemifield (four functions for each participant). The average ± SD map scaling factor (*c*, from Equation 3) for these functions in participants with albinism (*n* = 20) was 0.058 ± 0.018. Because each eye was stimulated separately, the cortical mapping functions from each eye were compared within each hemisphere by comparing the map scaling factors. The functions from the right and left eyes were not significantly different, paired *t*-test: *t* = 0.65, *df* = 9, *p* = 0.47, so the two map scaling factors from the same hemisphere were averaged together. When these average map scaling factors for participants with albinism were compared across hemispheres, there was no significant difference between the right and left hemispheres, Wilcoxson matched-pairs test, *p* > 0.99.

The map scaling factors (*c* from Equation 3) were then compared between controls and participants with albinism (Figure 6A vs. 6C). Because there were no differences between hemispheres (for both groups) or between eyes (for participants with albinism), the map scaling factors from all functions in each participant were averaged together, yielding a single overall estimate of the cortical mapping function for each participant. When the average map scaling factors for control participants, *n* = 5, mean ± SD = 0.0587 ± 0.0048, were compared with those for participants with albinism, *n* = 5, mean ± SD = 0.0579 ± 0.0158, there was no significant difference, Mann-Whitney *U* test: *U* = 9, *p* = 0.55.

The fitted cortical mapping functions were then used to derive the CMF and CMF scaling factor (1/*c*) for each task in each participant (see Equation 4 in Methods). The resulting functions for controls and participants with albinism are shown in Figure 6B and 6D, respectively. The CMFs for control participants were modeled separately for each hemisphere (*n* = 10), and the average ± SD value for 1/*c* was 17.33 ± 2.26. For participants with albinism, CMFs were modeled separately for each eye and each hemisphere (stimulated by the contralateral visual field, *n* = 20), and the average ± SD value for 1/*c* was 18.94 ± 6.01. These CMF scaling factors (1/*c*) were then averaged together for each participant (as described elsewhere in this article when comparing cortical mapping functions). When the CMF scaling factors were compared between control participants, *n* = 5, mean ± SD = 17.33 ± 1.24, and participants with albinism, *n* = 5, mean ± SD = 18.94 ± 4.77, there was no significant difference, Mann-Whitney *U* test: *U* = 9, *p* = 0.55. However, the CMF scaling factor was more variable in albinism relative to controls, *F* test: *F* = 14.79, *dfn* = 4, *dfd* = 4, *p* = 0.023. This finding is evident in Figure 6B versus 6D (in Figure 6D, the dashed gray line is the average of the fitted curves from control participants, and the shaded gray area represents the values within 1 SD of the average).

The size of V1 is known to directly impact the magnitude of estimated CM, although the shape of the function does not change significantly (Sereno et al., 1995). To verify that the trend toward a smaller V1 surface area in participants with albinism did not also affect the comparison between control and albinism groups, the map scaling factor in each task was compared with the V1 surface area of the corresponding hemisphere. The map scaling factors were not significantly correlated with V1 surface area in the corresponding hemispheres in either control, *r*^2^ = 0.18, *p* = 0.22, or albinism groups, right eye: *r*^2^ = 0.33, *p* = 0.14; left eye: *r*^2^ = 0.25, *p* = 0.20.

**Figure 7.**
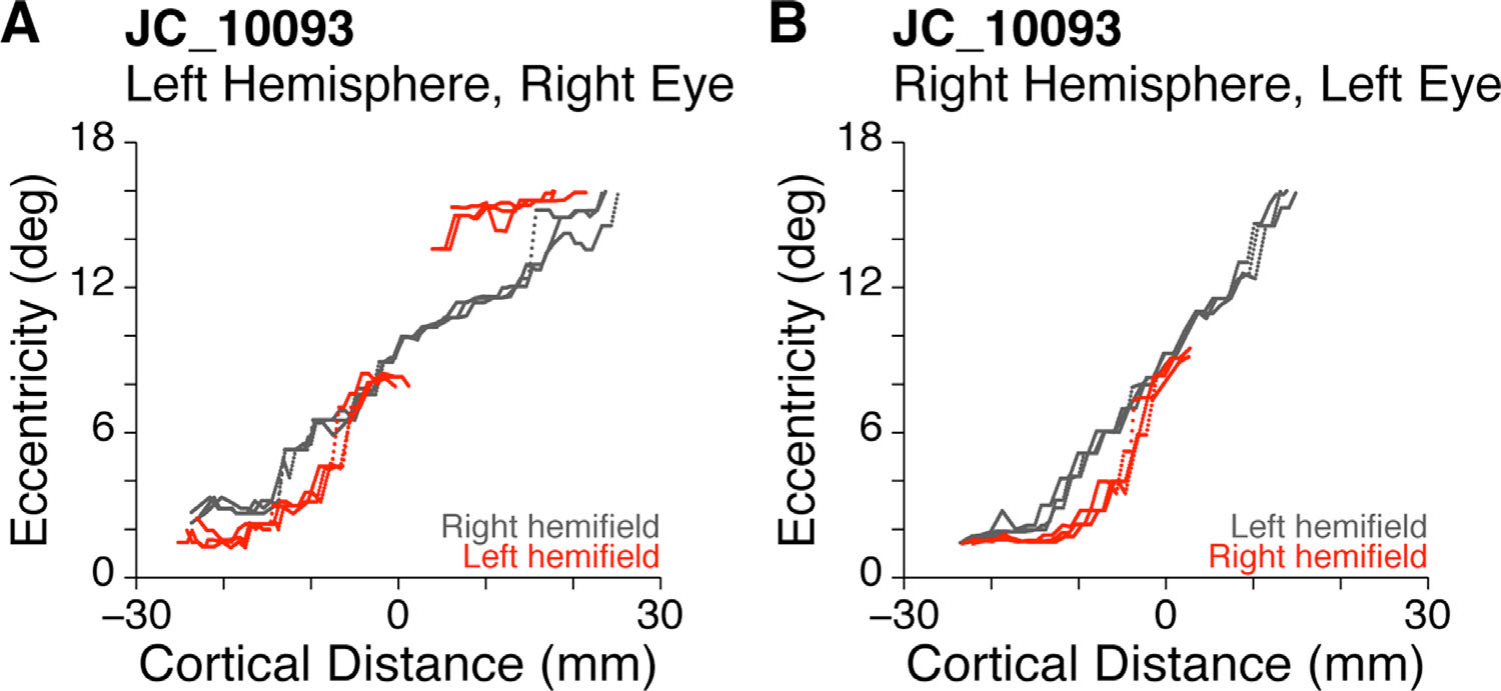
Comparison of empirical cortical mapping functions for normal contralateral (gray) versus aberrant ipsilateral (red) hemifield stimulation in the left and right hemispheres of participant JC_10093. The red and gray data are from the same physical ROIs yet are distinctly different.

As mentioned elsewhere in this article, all participants with albinism had aberrant ipsilateral hemifield representations superimposed on the normal representation of the contralateral visual field (Figure 4C). For most participants, this ipsilateral activation was highly truncated compared with the contralateral activation. However, two participants had at least one hemisphere with sufficient ipsilateral activation to compare the aberrant activation to the “normal” activation within the same hemisphere. Figure 7 shows the comparison between aberrant (red) and normal (gray) hemifield stimulation in one of these unique participants, JC_10093. For this participant, the eccentricity mapping from the two hemifields differed significantly even though both curves arose from precisely the same voxels. In contrast, Figure 5 (JC_10093) compares activation from the same ROIs when activated by right (red) versus left (blue) eyes using the dominant contralateral hemifield stimulus. In Figure 5 the red and blue curves are quite precisely matched.

### Empirically based versus cone- and RGC-predicted models of CM

**Figure 8.**
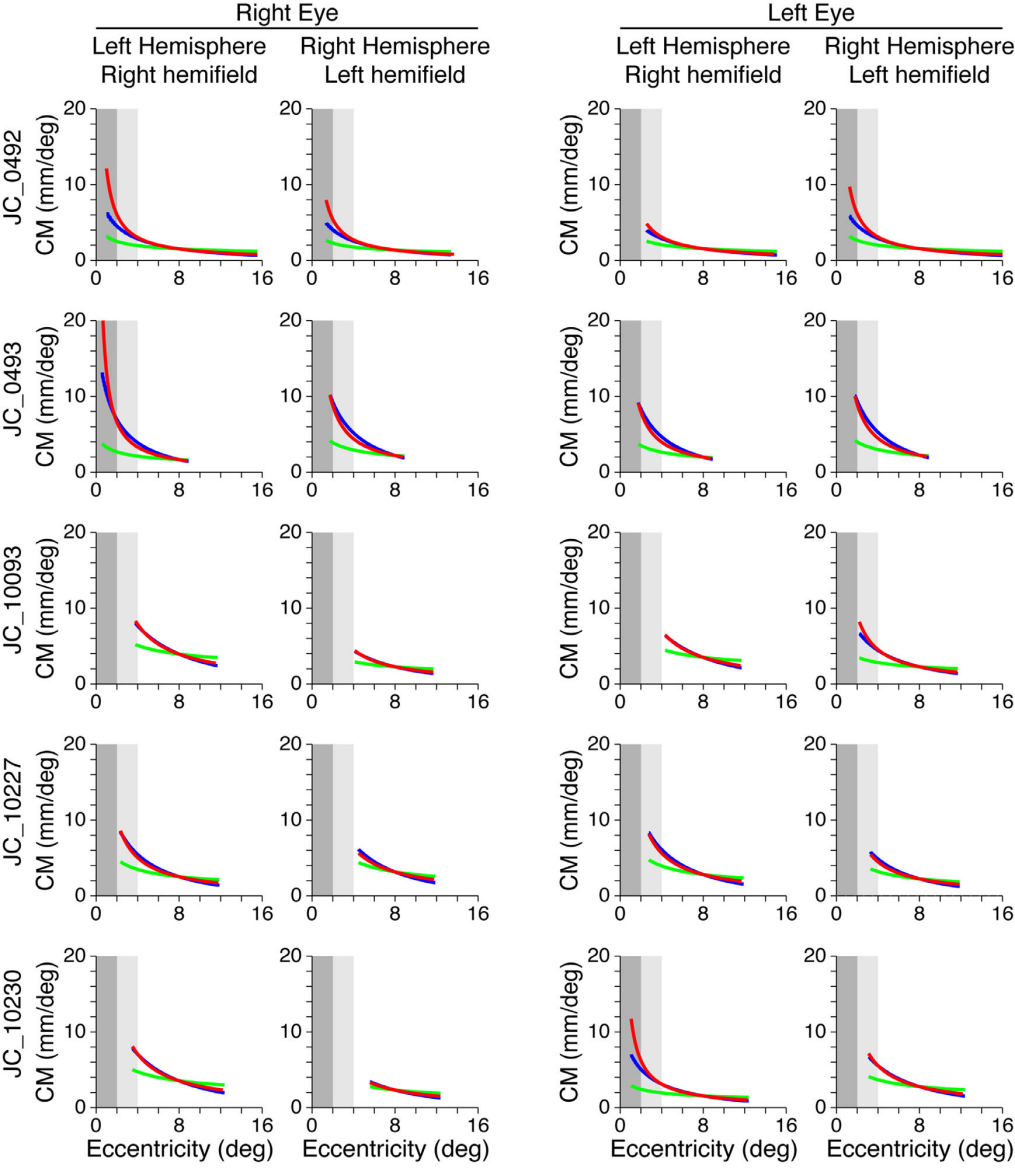
CMFs based on empirical data (red), cone density predictions (green), and RGC predictions (blue) for participants with albinism. To aid comparison, eccentricity ranges from 0° to 2° and 2° to 4° are shaded in dark and light gray, respectively.

**Figure 9.**
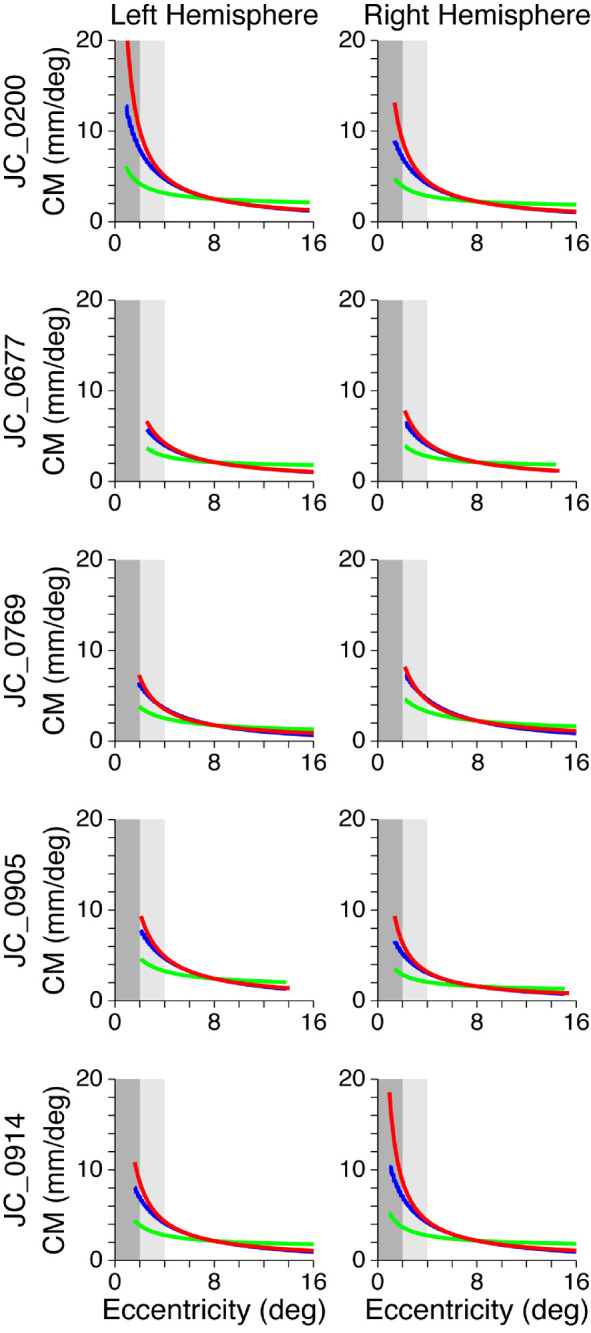
CMFs based on empirical data (red), cone density predictions (green), and RGC predictions (blue) for control participants. To aid comparison, eccentricity ranges from 0° to 2° and 2° to 4° are shaded in dark and light gray, respectively.

Figure 8 shows empirically derived CMFs (red) for each participant with albinism along with a predicted CMF based on cone density alone (green) and on RGC density (blue). Results for control participants are shown in Figure 9. As outlined in the Methods, the cone density and RGC predictions have been intentionally aligned to the empirically based curves at 8° eccentricity to facilitate comparison of the shapes of the curves rather than their absolute differences (which are sensitive to an arbitrary scaling factor). It is clear in all hemispheres for which the data extend to within 2° of the center of gaze (Figure 8, dark gray shaded region) that the empirically measured CM (red) increases far more rapidly than is predicted by cone density alone (green), and this is also the case (albeit to a lesser extent) between 2° and 4° eccentricity (Figure 8, light gray shaded region). In contrast, the CM predicted by RGC density (blue) is a much better match for the empirically measured CM, although the RGC predictions and the empirical measurements tend to diverge within 2° eccentricity. This is true for both albinism (Figure 8) and control groups (Figure 9).

To determine whether cortical organization was correlated with cone density, the cortical mapping functions and CMFs were also compared with each participant's peak cone density. Because the cone density was measured in only one eye in each participant, only the cortical functions based on activation by the imaged eye (i.e., the right eye in all participants except JC_10230; see Table 1) were included in this analysis. When the average map scaling factor (*c*) and the CMF scaling factor (1/*c*) from both hemispheres were compared with cone density, neither the map scaling factor nor the CMF scaling factor was significantly correlated with peak cone density in either control participants, *c*: *r*^2^ = 0.0062, *p* = 0.90; 1/*c*: *r*^2^ = 0.0080, *p* = 0.89, or in participants with albinism, *c*: *r*^2^ = 0.16, *p* = 0.51; 1/*c*: *r*^2^ = 0.22, *p* = 0.43.

## Discussion

### Aberrant retinotopy in albinism

The results of this study are largely consistent with previous reports of highly aberrant retinotopic organization in visual area V1 of participants with albinism (Creel, Witkop, & King, 1974; Dorey, Neveu, Burton, Sloper, & Holder, 2003; Hoffmann et al., 2003; Schmitz et al., 2004). This study significantly extends those findings to include an analysis of individual variability and a quantitative account of CM compared with individual retinal cone density measurements. Aberrant retinotopic organization was apparent in all participants with albinism and consisted of superimposed representations of opposite hemifield representations within the same hemisphere. This is due to ganglion cell projections from the temporal retina, which normally synapse in the ipsilateral hemisphere, but in albinism decussate to the contralateral hemisphere (Creel et al., 1978; Hoffmann et al., 2003). The aberrant (ipsilateral) hemifield activation was most prominent in the hemisphere contralateral to the eye being stimulated. This aberrant activation typically extended to approximately 5° to 6° from the center of gaze, but the extent varied considerably across the albinism group. This agrees with previous studies that found significant variation in the left–right margin of aberrant decussation among individuals with albinism (Dorey et al., 2003; Hoffmann, Lorenz, Morland, & Schmidtborn, 2005; von dem Hagen et al., 2007).

When the surface area of V1 was measured in this study, participants with albinism had modestly decreased surface area relative to control participants, but this difference was not statistically significant (Figure 3). Changes in occipital gray matter volume that were reported in previous studies of albinism were highly localized to the occipital pole (Bridge et al., 2014; von dem Hagen et al., 2005); thus, they would be unlikely to significantly affect measurements of V1 surface area as a whole. However, participants with albinism are also known to have decreased optic nerve, optic chiasm, and optic tract size (Ather et al., 2019; von dem Hagen et al., 2005), which may reflect a decrease in retinal afferents from the foveal region where cone density is reduced (Wilk et al., 2014). In normal visual system development, the V1 surface area is thought to be correlated with optic tract size (Andrews et al., 1997). Thus, the apparent lack of correlation between previous reports of a decreased optic tract size in albinism and our measurements of nearly normal V1 surface area in albinism suggests that unique factors involved in the development of retinocortical projections in albinism may preserve cortical space despite a decreased number of retinal afferents. This finding might suggest that the decreased number of retinal afferents found in albinism spread out to occupy a comparable area of cortical space as the more numerous retinal afferents found in control participants. Whether such variation in innervation density would occur in the retina, thalamus, V1, or incrementally at all levels is unclear.

### Variability in retinotopic organization in albinism

In the albinism group, we noted marked variations in the overall pattern of retinotopic organization that have not been fully appreciated previously. These features include both the continuity of the retinotopic maps and the relative area of V1 activated by the central versus the peripheral visual field.

Qualitatively, the area of cortical activation corresponding with the central visual field in participants with albinism often seemed to be decreased as compared with control participants, with a corresponding increase in cortical area activated by the perifovea (red vs. green regions in Figure 4 and Supplementary Figure S1). This finding may be partly due to the visual task used for retinotopic mapping in participants with albinism, because the smallest annulus in the expanding ring stimulus did not include the foveal center. Rather, the center of fixation was covered by the round fixation marker that had a radius of 0.8° and appeared randomly to control for attention. Although the central activation might have been improved by using a contracting ring or drifting bar stimulus (Dumoulin & Wandell, 2008), Ahmadi et al. (2019) recently used a drifting bar stimulus for retinotopic mapping in albinism and also noted decreased foveal activation. Moreover, the presence of robust foveal activation in several participants with albinism (e.g., JC_0492 and JC_0493) indicates that our stimulus was capable of evoking strong perifoveal cortical activation in at least some of our participants with albinism. Another concern was that variation in the continuity of the retinotopic maps could be due to reduced participant alertness during imaging. However, we explicitly monitored alertness by self-report after every fMRI scan. Only one participant with albinism (JC_0493) reported alertness levels below 3 (on a scale from 1 to 5, where 5 is most alert), yet this participant had some of the most robust activation patterns. We also considered the possibility that, for some participants, a decreased foveal representation might reflect disproportionate spatial distortion and signal dropout at the occipital pole owing to magnetic field inhomogeneities induced by the transverse sinus (Winawer, Horiguchi, Sayres, Amano, & Wandell, 2010). A close inspection of the sinus anatomy and blood oxygenation level-dependent fMRI images did not reveal any obvious differences in the participants who had the most reduced foveal representations. Likewise, at the group level, no obvious vascular differences could be detected between participants with albinism and control participants. In sum, we do not believe that the decreased foveal representation in some participants with albinism is likely to be artifactual.

Our observation that central visual field activation was decreased in albinism is consistent with a previous study that reported a similar finding (Schmitz et al., 2004). Although this decreased central activation (relative to peripheral activation) seemed to correlate with decreased peak cone density in four of five participants with albinism (all except JC_10230), an explicit statistical test for correlation between V1 surface area within 4° and peak cone density was not significant. This indicates that factors other than cone density (e.g., the divergence or convergence of retinocortical projections) may also contribute to the relative sizes of the cortical representations of the central versus peripheral field.

### CM: Albinism versus controls

Despite grossly aberrant hemifield organization, the cortical mapping functions for participants with albinism were not (on average) significantly different from those for control participants (Figure 6C; albinism curves shown in magenta, average control function shown in gray). As shown in Figure 6B, our control data also match those of Sereno et al. (1995) and Engel et al. (1997). This similarity between controls and participants with albinism was also evident when the fitted cortical mapping functions were used to model the CMF in each hemisphere (Figure 6D). It is important to stress that, for our CM analysis, we took particular care to avoid potential interactions and confusions of different eye and hemifield conditions by stimulating each eye and visual hemifield separately in different scan runs. This strategy was designed to prevent single voxels from being activated by two different hemifields or eyes at the same time, thereby allowing us to assess CM independently for each eye and hemifield condition. The lack of significant differences in CM between controls and participants with albinism allows us to reject our initial hypothesis that decreased cone density in albinism would necessarily lead to a decreased CM. Instead, some of the hemispheres of participants with albinism tended to have a greater CM in peripheral regions of the visual field compared with the control group (Figure 6D, magenta curves outside the gray underlay representing controls). This trend in CM is not explained by variation in the V1 surface area, which did not differ between our albinism and control groups (and even trended in the opposite direction). This finding indicates that, in albinism, retinal cone density does not determine the size of the retinotopic representation in V1. This finding contrasts with the hypothesis that has previously been suggested for normal controls (Dougherty et al., 2003). Rather, it suggests that the cones that are present (along with their downstream synaptic partners) capitalize on the entirety of the cortical space available to them. In this scenario, it is possible that the amount of cortical space devoted to V1 (and other visual areas) is heavily influenced by postreceptoral factors that may be unique to albinism (or similar genetic conditions).

It is also notable that, for one of the participants with albinism (i.e., JC_10093), the cortical eccentricity mapping functions for the normal and aberrant fields had local zones in which the eccentricities differed markedly, despite being represented by exactly the same cortical voxels (Figure 7). This indicates that the two hemifield representations are not always precisely in mirror image register, which, in turn, suggests that more subtle wiring anomalies can occur in addition to those related to a shift in the line of left–right decussation at the optic chiasm. Although one might suppose that these more subtle errors simply represent random variations, it is important to appreciate the precision of same-hemifield overlap in normal controls that gives rise to the systematic and precise computations of retinal disparity responsible for stereopsis, which can be precise to within fractions of a degree. Moreover, the cortical eccentricity mapping functions from this participant were highly consistent across independent individual scans (data not shown). In albinism the aberrant left–right hemifield registration seems to be precise in some places, but can be misregistered by several degrees in other locations. Whether the lack of precise registration between opposing hemifield representations in albinism has any functional significance remains unclear.

### CM: Empirical versus cone- and RGC-based predictions

The relationship between CM and cone density was explored further by comparing the empirically derived CMF models with two predicted CMF models: one based on cone density alone, assuming that each cone was allocated an equal amount of cortical space in V1, and one based on RGC density, assuming that each RGC receptive field was allocated an equal amount of space in V1. All participants—both controls and those with albinism—showed a greater increase in CM near the center of the visual field than would be predicted based on cone density alone, but an RGC-based prediction was much closer to empirical measurements (Figures 8 and 9). When the empirical and predicted CMFs are compared in the central-most regions (see the shaded areas in Figure 8), it is evident that both the magnitude and slope of the empirical functions (red) are greater than the cone-based predictions (green). This finding is also true for the RGC-based predictions (blue), but there is less divergence between the slopes of this prediction and the empirical data, and any differences that are present are only apparent within the central 2°. Note, however, that the empirical data within the central 2° (dark gray zone) are marginal owing to limitations of the stimulus (see Methods), and this finding was particularly true for two of the participants with albinism (JC_10093, JC_10227). This difference is important because one might expect that the significant loss in cone density observed in albinism relative to control participants would lead to markedly decreased CMFs. However, the greatest differences in cone density are observed primarily at the fovea and do not extend beyond 2°, where our more reliable CM data begin. This finding might also account for the failure to observe major differences in CMF between participants with albinism and controls.

Our observation that empirical CM values exceed the cone-based predictions might be explained by a number of factors. It is known that there is greater convergence of cones onto peripheral RGCs compared with central RGCs (Curcio & Allen, 1990; Dacey, 1993; Dacey & Petersen, 1992), which would exacerbate cone-based differences in central versus peripheral magnification. This eccentricity-dependent RGC sampling of cones is clearly an important factor in predicting CM, because our RGC-based predictions were much closer to empirical measurements than our cone-based predictions. It is important to note, however, that our RGC-based CM predictions assume that the cone:RGC ratio in albinism is similar to that in controls. Recent evidence indicates that the normal circuitry between cones and RGCs may be disrupted in individuals with foveal hypoplasia (Dacey, 2018), so it is unknown whether this model accurately reflects the relationship between these cells in this population.

Moreover, in most participants who showed clear cortical activation within 2° of the fovea (JC_0200, JC_0914, JC_0492, and JC_10230), there remained small disparities between the empirical CMF and the RGC-based predictions of CMF near the fovea. This finding may be due to the fact that RGCs with receptive fields near the center of the visual field are thought to project to more cortical space than peripheral RGCs (Azzopardi & Cowey, 1996; Popovic & Sjöstrand, 2001); however, this hypothesis is debated (Wässle, Grünert, Röhrenbeck, & Boycott, 1990). To examine the representation of RGCs in V1, one must consider both the convergence and the divergence of the RGC projections onto LGN neurons and the divergence of LGN projections onto layer four of V1. Indeed, Azzopardi and Cowey (1996) argue that the final V1 CMF is the result of successive expansion of the foveal representation both in the LGN and in the cortex, and Stevens (2002) argues that the number of neurons in V1 increases by the 3/2 power of the number of input neurons from the LGN. The stage between the LGN and the cortex was described in detail by Connolly and Van Essen (1984) for the macaque monkey, and they found that “The total number of cortical neurons per LGN neuron is about 130 on average, but it extends over approximately a tenfold range, from less than 100 in the far periphery to nearly 1,000 in the fovea.” Whether this tenfold difference in divergence is also true for humans with or without albinism is unknown, but it is likely to be a major factor in determining the resulting CM. Clearly, a comprehensive, quantitative account of the neural basis of CMF must await more extensive estimates of convergence and divergence versus eccentricity at each stage of the retinostriate hierarchy.

### Behavioral predictions

Given previous observations that human visual acuity is normally correlated with CM (Duncan & Boynton, 2003), the similarities in CMFs between controls and participants with albinism in this study might predict that parafoveal and peripheral visual acuity would be similar in albinism to that in normal controls. A previous study of acuity in albinism found that central visual acuity in albinism was decreased, but that peripheral visual acuity was similar to normal controls, which supports this prediction (Wilson et al., 1988). However, that study only measured acuity at the center of gaze and at 10° inferior, so it is currently unclear at what eccentricity acuity approaches normal levels in albinism. Although our measurements of CM were limited to 2° to 16° (i.e., they did not extend to the fovea), the empirically derived CMFs in participants who had the most extensive central representations seem to remain within normal limits (see Figure 6D). If this trend continues all the way to the fovea, it would indicate that the CMFs may not necessarily correlate with the decreased central visual acuity typically observed in albinism. Moreover, as in amblyopia (Clavagnier et al., 2015), participants with albinism may have a different cortical correlate of visual acuity, such as population receptive field size rather than CM per se. However, the variability in cortical organization shown in this study indicates a role for plasticity in modifying the functional relationship between retinal and cortical structures. More detailed studies of visual acuity are needed in this population along with targeted investigations of the cortical foveal confluence to further explore the etiology of the visual acuity deficits in albinism.

### Limitations and future directions

One of the primary limitations of this study is the small sample size. Both the rarity of albinism and the prevalence of moderate to severe nystagmus within this population serve as barriers to the recruitment of large numbers of participants with albinism who are good candidates for fMRI. However, even in the small cohort presented here we observed significant variability between individuals with albinism, both in retinotopic organization and in CM. This finding is consistent with previous studies that have shown significant variability in retinal phenotypes among individuals with albinism, particularly in the severity of foveal hypoplasia (Kruijt et al., 2018; Thomas et al., 2011) and in peak cone packing density (Wilk et al., 2014; Wilk et al., 2017). Indeed, our group previously performed a detailed analysis of the retinal structure of the participants presented in this study (Wilk et al., 2014; Wilk et al., 2017), and this analysis was used to intentionally select participants with albinism for fMRI imaging who represented a broad spectrum of retinal structure (for cone densities, see Figure 2). The findings of this study only increase the importance of measuring cortical phenotypes in more individuals to determine the full extent of phenotypic variability. Additionally, it is possible that phenotypic variability in albinism may be correlated with the subtype of albinism and/or specific alleles that each individual carries, but a larger participant cohort is needed to address this question definitively. Learning more about genotype/phenotype correlations in albinism will be essential for guiding clinical diagnosis and for developing interventional therapies.

Another potential concern is that nystagmus and eccentric fixation are common in albinism and might adversely affect the retinotopic maps. Although nystagmus can introduce noise into the fMRI signal, it is less likely to systematically alter the spatial properties of a retinotopic map (Baseler et al., 2002). Moreover, BCEA values comparable with those reported here for all but one participant with albinism do not seem to affect the CM in other populations with unsteady fixation (Clavagnier et al., 2015). Eccentric fixation, in contrast, can affect the shape and symmetry of cortical mapping functions (Baseler et al., 2002). However, the cortical mapping functions that we observed were notably symmetric between hemispheres, suggesting that eccentric fixation is unlikely to have had a significant effect on the data presented here.

Finally, for many participants the cortical mapping data did not extend to the central 2° of the visual field, which was largely due to limitations of the visual stimulus (see Methods). This lack of foveal data precluded any definitive conclusions about the relationship between cone density and CM at the fovea, which is where the greatest difference between albinism and normal vision might be expected. Future investigations may be more successful by using a drifting bar stimulus instead of an expanding ring/rotation wedge stimulus, because the drifting bar has been shown to provide greater precision at the fovea (Dumoulin & Wandell, 2008).

## Conclusions

Albinism provides an excellent model in which both peripheral and central effects of genetic mutations can be explored quantitatively. This study confirms previous findings of abnormal retinotopic organization in albinism and expands on those findings by showing that there is greater diversity in retinotopy across individuals with albinism than previously appreciated. Moreover, these variations do not correlate with variations in retinal cone density. CM outside the fovea is not significantly different in albinism than in normal controls and is greater in both groups than is predicted by cone density alone.

Overall, our results show that the pattern of retinocortical miswiring that has previously been ascribed to aberrant left–right decussation at the optic chiasm is significantly more complex and varied than previously thought. Whether this additional complexity occurs at the retina, the optic chiasm, or represents additional connectivity changes downstream is unclear. Future models of the development of visual pathways in albinism must account for both the observed changes in cone density and the absence of changes in CM.

## Supplementary Material

Supplement 1
