# Supplementary material for: The relationship between retinal cone density and cortical magnification in human albinism
Source: J Vis. 2020 Jun 16;20(6):10. doi: 10.1167/jov.20.6.10 (PMC7416892; doi:10.1167/jov.20.6.10)
Supplement: Supplement 1 [file jovi-20-6-10_s001.pdf]

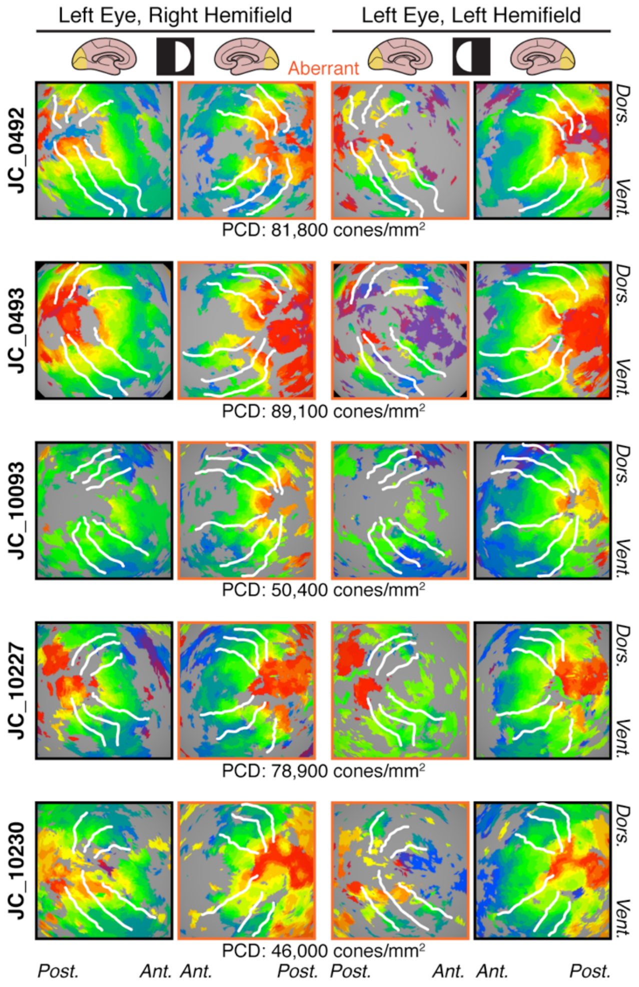

Supplementary Figure S1: Retinotopic maps of visual field eccentricity for left eye viewing condition in subjects with albinism. Visual stimulus and color coding as shown in Figure 4A. Retinotopy patterns outlined in orange are aberrant ipsilateral hemifield representations. Peak cone densities for each subject with albinism indicated below each row. Maps are displayed on spherically-inflated cortical surface models. Visual field stimuli are indicated by white semicircle symbols at head of respective columns. White lines mark dorsal and ventral boundaries of V1/2/3 based on polar angle data (cf. Figure 1). PCD = peak cone density; Dors. = dorsal; Vent. = ventral; Ant. = anterior; Post. = posterior.
